# Nanotechnology in drug delivery: the need for more cell culture based studies in screening

**DOI:** 10.1186/1752-153X-8-46

**Published:** 2014-07-16

**Authors:** Aminu Umar Kura, Sharida Fakurazi, Mohd Zobir Hussein, Palanisamy Arulselvan

**Affiliations:** 1 Laboratory of Vaccine and Immunotherapeutics, Institute of Bioscience, Universiti Putra Malaysia, 43400 Selangor, Malaysia; 2 Faculty of Medicine and Health Science, Pharmacology Unit, Universiti Putra Malaysia, Selangor, Malaysia; 3 Materials Synthesis and Characterization Laboratory, Institute of Advanced Technology, Universiti Putra Malaysia, Selangor, Malaysia

**Keywords:** *In vitro*, Animal studies, Nanodelivery system, Toxicity and bio distribution

## Abstract

Advances in biomedical science are leading to upsurge synthesis of nanodelivery systems for drug delivery. The systems were characterized by controlled, targeted and sustained drug delivery ability. Humans are the target of these systems, hence, animals whose systems resembles humans were used to predict outcome.

Thus, increasing costs in money and time, plus ethical concerns over animal usage. However, with consideration and planning in experimental conditions, *in vitro* pharmacological studies of the nanodelivery can mimic the *in vivo* system. This can function as a simple method to investigate the effect of such materials without endangering animals especially at screening phase.

## Introduction

Nanodelivery system (NDS) is a branch of Nanomedicine characterized by controlled, targeted and sustained drug delivery ability, a limitation and drawback that is currently limiting conventional drug delivery system especially in drug delivery to tight areas like the brain [1]. NDS, due to their small sizes usually below 100 nm and unique physico-chemical and biological properties, are now becoming the favourable system in drug delivery and imaging system covering wider ailments including cancers and central nervous pathologies [2,3]. Drugs susceptible to enzymatic degradation and/or pH destabilization can be incorporated into this delivery system, it also offer them with additional possibilities of targeted and controlled release potential [4]. However to achieve these foreseeable advantages offered by nanodelivery systems, challenges which include developing toxic-free system, improved biocompatibility, effective drug loading, proper targeting, transport and release ability must be ascertained [1]. Biocompatibility and bio-distribution are part of the key to success for drug development in achieving its aim, consequently more *in vitro* and *in vivo* study needed to achieve the desired characteristics.

However, with changes and improvement in drug delivery via NDS comes a price (possible toxic effect), the evaluation of which is important before any biological application can be introduce. In 2004, the term nanotoxicity was coined; referring to the study of the potential toxic impacts of nanoparticles on biological and ecological systems [5]. The field arose due to concern over the growing field of nanotechnology and the potential health effects of nano-materials, especially to humans. The low soluble or insoluble type nano-material capable of passing through various defence systems because of their tiny size are of the greatest concern [6]. The toxicity and bio-distribution of these delivery systems could be influenced by the synthetic process; coating materials; particle sizes and or route of administration [7,8]. Hence, toxicity and distribution studies should target these to evaluate the potential of NDS in drug delivery.

Animals have been in the forefront of chemical toxicity test, including drugs intended for human consumption [9]. In 2005, 20% of the over 10 billion euro spent on animal experiments worldwide and 100million animals used were in toxicity studies [9]. Although, animal usage is a part of the legislation before chemical usage is allowed for both life and environmental protections [10]. The values of the obtained results are sometimes challenged with respect to transfer to humans, extreme doses application during the studies and a time a false positive correlation are made with respect to the low toxicity of most of the tested chemicals [10]. Animal based-studies are generally costly in term of money and time, plus ethical issues associated with it, these reasons make tissue and cell-based exposure studies very useful for toxicity screening of new compounds (nanoparticles inclusive). Therefore, human as well as other animal tissue/cells will continue to serve as an alternative (*in-vitro*) test methods. The application of which may provide endpoints result, which are directly associated with specific organs. Nevertheless, it is well acknowledged, especially in the European community that cell and tissue cultures will not be able to replace animal tests completely [11]. This is mainly due to the complexity of the human and the short lifetime of culture, negating for the assessment of chronic toxicity needed before human application [11].

In the past, cytotoxicity study used few survival mechanisms following exposure to the chosen chemicals, mechanism like mitochondrial activity, membrane damage and enzymes leakages into the culture media were studied. However, advances were made over the years, which resulted in cell usage for gene analysis in toxicity, microscopic changes in cell cytoskeleton due to toxicity among others [12-15]. This review aimed at reviewing some of those methods used in assessing nanomaterial toxicity using cell/tissue culture technique, with emphasis on their advantage over entire animal usage, especially during screening processes.

## Review

### Nanodelivery systems effects in relationship to dose and time

Among the barrage of methods used in the NDS toxicity assessment is the whole cell quantification following exposure to certain concentration of the expected toxic nanomaterial as compared to the untreated cell [2,3]. These methods have the advantage of conveying the actual number of viable cells, an increase (cell proliferation) or decrease (cytotoxicity) in comparison to control (untreated cells). In some instances, they measure the activity of viable cells in the treated sample and the control, indirectly counting live and dead cells [2,3].

Trypan blue is an example of quantitative dye exclusion assay. In this experiment, cells are treated with NDS or any chemical/drug for a desired period, then stained with trypan blue to observe for cell proliferation or cell’s dead [16]. The dye is called an exclusion diazo dye, which is taken up by dead cells only, but excluded, by viable cells [16]. The cells are viewed at higher magnification using a light microscope, where unstained cells reflect the total number of viable cells recovered from a given treatment. The toxicity potential of zinc-layered hydroxide (ZLH) and its corresponding cetirizine nanocomposite (CETN) were tested on normal Chang liver cells after 24 hours of treatment using the trypan blue stain; it was done to show dose-dependent toxic effects of the nano-carrier (ZLH) as compared to the corresponding intercalated counterpart (CETN) [17]. Other nanomaterial tested using this method included but not limited to zinc oxide (ZnO), copper oxide (CuO) and multi wall carbon nanotubes (MWCNT) [12].

Stress exposure in the form nutrient deprivation or drugs induced toxicity, could lead to necrotic or apoptotic death at the cellular level [18]. Propidium iodide and acridine (AOPI) are examples of double dye stain; they can be used to study the apoptotic and necrotic cell death. Viable cells stain green, while apoptotic cells stain green and shrunken with condensation of the nucleus, the necrotic cells stain red [18]. These morphological changes will appear under fluorescence microscope with AOPI stain. Propidium iodide (PI) intercalates into double-stranded nucleic acids of dying or dead cells as it can penetrate damaged cell membranes. It is excluded by viable cells, but acridine orange (AO) can penetrate intact membrane to stain viable cells [18]. Copper oxide nanoparticles were shown to induce apoptosis and subsequent cell death, in human skin keratinocytes, utilizing the AOPI staining technique [13]. Apoptosis is generally regarded as an active or programmed form of cell death. It is the preferred cell death; it allows the normal immune system to get rid of the demise cells and tissues from the system [14]. In contrast to this method of cell death is the necrotic method, also referred to as uncontrolled or pathological cell death. The two systems, are not exclusively separated as they may happen at the same time on the same tissue, especially where different concentrations were used [14].

As stated earlier apoptotic cells stands the chances of being engulfed by neighbouring immune cells, but necrosis may lead to a secondary effect especially where immunity has weakened and or inefficient. These and other double dye studies are capable of screening NDS and other compounds at the cellular level for a possible cell death method before endangering animal or human lives. Using this technique, silver nanoparticle was shown to have both apoptotic and necrotic effects on breast cancer cells (MCF-7) in a dose dependent fashion [19]. Doses below 50 μg/mL demonstrated apoptotic death and above 80 μg/mL caused more of necrotic cell death. Not just killing the cancer cell is important, but also what happen after cancer cell death. The proceeding secondary reaction due to the toxic substances release to the surrounding normal cells by the dying cancer cells could be more deadly than cancer itself [20]. A clinical syndrome called tumour lysis syndrome seen in the case cancer cells lysis, could be reduce if apoptosis is induce more in cancer treatment rather than necrosis. Thus, results like this one could be handy in deciding doses to be use during animal and subsequent clinical trials.

### Cell functions and membrane integrity

Cellular metabolism and cell membrane integrity as functions of living cells were used in the past to assess drug and other chemical’s toxicity. Now they are in use for NDS screening [21]. Proliferation assay like MTT (3-(4,5-dimethylthiazol-2-yl)-2,5-diphenyltetrazolium bromide) and neutral assay are typical examples of functional assessment used in toxicity and or efficacy studies. They are rapid and convenient in determining viable cell number in proliferation or cytotoxicity studies. While lactate dehydrogenase (LDH) assay is, an example of cell membrane integrity study commonly applied in the study of nanoparticle toxicity, as well as other drugs and chemicals intended for human or environmental uses [21]. LDH is a cellular enzyme that is release into the cytoplasm upon cell lysis. The assay, therefore, can be used to measure membrane integrity. Principally the assay work by converting lactate to pyruvate through oxidation by the LDH, subsequently Pyruvate reacts with the tetrazolium salt to form formazan, water-soluble formazan dye that can be detected by a spectrophotometer [21].

In the case of MTT, the assay is dependent on the reduction of tetrazolium salt MTT (3-(4,5-dimethylthazol-2-yl)-2,5-diphenyl tetrazolium bromide) by mitochondrial dehydrogenase of only viable cells to form a blue formazan product [15]. Neutral red on the other hand is an uptake viability assay [22]. Principally it works by the accumulation of the dye in the lysosomes of uninjured cells.

Utilizing some of the above-described assays, doses and time-dependent effect of different types of nanomaterial was evaluated to stimulate and estimate possible *in vivo* effect [12,13,17]. In most instances, 24-72h period was employed to determine these effects, which will have been days to weeks if similar effects were to be determined in animals. For example, eighty experimental animals of both sexes were used to study the possible toxicity of a synthesized titanium oxide nanoparticle of different sizes and over three weeks period used [23]. The tissues collected from these animals undergoes several days of processing before a decision emerged as to either the titanium oxide nanoparticle used where toxic or not [23]. Above stated assays will have been a better choice in screening these nanoparticles for their likely toxicity, and where toxicity exists, modification can be made at the level of synthesis with the view to address what causes the toxicity. Satisfactory results obtained from these *in vitro* assays will help in designing a successful *in vivo* study, limiting wastage in lives, money and time.

### Oxidative stress studies of nanodelivery systems

*In vitro* assays such as nitric oxide (NO) assay (Greiss reaction) are used as a measure of free radical production following exposure to NDS or any oxidative stress inducing drugs [24]. This measurement provides a surrogate marker and quantitative indicator of nitric oxide production due to a nano-delivery treatment of particular cell line or tissue. Other oxidative stress assays such as glutathione reductase (GR), reactive oxygen species (ROS) are also valuable in assessing oxidative stress potential of nano-delivery systems. Cytotoxicity and free radical assessment via nitric oxide analysis is becoming very curial [25]. This is so due to the growing evidence, that high concentrations of nitric oxide (NO) in the brain might be involve in a variety of neurodegenerative diseases, of which Parkinson disease is one of them. Others are Alzheimer's, cerebral ischemia and epilepsy [25]. These and other related oxidative stress markers associated with different diseases, including cancers could be predicted with ease using cell culture techniques [26]. The timing of which could be considered relatively short when compared to animal studies of similar aim. Within 24h of exposure, titanium oxide nanoparticle demonstrated a dose related increase in reactive oxygen species from normal human bronchial epithelium (BEAS-2B) [27]. In the same study, a relationship was established between production of ROS and glutathione reductive enzymes (GSH) depletion. Recently, an important development was demonstrated in the treatment of cardiomyopathy with cerium oxide (CeO2) nanoparticles [28]. This group of nanoparticles were shown to modulate oxidative stress in transgenic mice with cardiomyopathy through free radical scavenging activity. The promising result was generated from animal model study, but the pre-requisite and preliminary findings were obtained from cell culture models (29. 30). Where, CeO2 nanoparticles was shown to rescued HT22 cells from oxidative stress-induced cell death [29], in another related study it was demonstrated to protect normal human breast cells from radiation-induced apoptotic cell death [30]. Here, *in vitro* studies predicted an outstanding animal result, negating the need for excessive animal usage. Thus, proper screening and designing of preliminary cell culture work will likely limit the number of animal to be use, indirectly cutting down cost and preserving ecological balance.

### Uptake mechanism and blood brain barrier delivery of drugs

The cellular uptake and delivery of nanoparticles across the blood brain barrier is another aspect of pharmacology where cell culture technology is of essence. With proper usage of available *in vitro* techniques, tedious procedure like micro dialysis, used in evaluating delivery of nanoparticles and other drugs to the brain can be minimize. Other molecular details like the receptor involve in nanoparticle transport across the BBB could also be explore using these models [31]. A group of scientist demonstrated the role of temperature and alternative receptor in transporting poly (methoxypolyethyleneglycol cyanoacrylate-co-hexadecylcyanoacrylate nanoparticle (PEG-PHDCA) to the brain [31,32]. These researchers used a relatively simple and cheap *in vitro* BBB model [31,32]. These cell culture models are becoming important tools in investigating drug delivery to the brain without necessarily torturing animals, especially during the initial screening phase [33,34]. Unlike the animal models, cell culture technology has the advantage of rapid evaluation in nanoparticle uptake mechanism, toxicity potentials and other related molecular mechanism needed in achieving drug delivery to the CNS.

The uptake and internalization of drugs intercalated into NDS can be studied with relative ease using *in vitro* cell models. An optical microscope and electron spin resonance (ESR) spectroscopic studies exemplifies this [15]. Where, the mechanism of uptake and internalization of iron oxide nanoparticles (IONs) into a brain tumour cell were shown [15]. A water dispersible iron oxide nanoparticle based formulation that was loaded with an anticancer agent also showed good cell penetration via an *in vitro* modelling [35]. The NDS (IONs) get internalize into the cell through cell membrane invagination, forming an early endosome that transformed into a late endosome within five minutes, then later a lysosome containing the particle and drug [18,36,37]. Entrance and uptake of paclitaxel-LDH nanoparticles bounded with FITC into cervical cancer was also studied using fluorescence and transmission electron microscope (TEM). The two demonstrated endocytosis as a means of cell entrance by the nanoparticles [9]. Demonstrating these, effects in animals require many lives to be sacrifice at intervals, added to which is a long and a lacklustre process needed before the result is acquired in most cases [38].

In the near future, thousands of these drug delivery vehicles may be synthesized for the treatment and diagnosis of CNS disease. Consequently, a BBB *in-vitro* cell culture models that closely resembles the *in-vivo* system, reflecting at least the characteristics "barrier", are in high demand. This is to minimise cost and other ethical issues attributed with *in-vivo* test systems. The *In vitro* BBB models are usually made-up of cerebral capillary endothelial or choroid plexus epithelial cells mostly of porcine in origin that closely resembles the animal brain system [33]. Interestingly, a reasonable correlation between, these *in vitro* models and the animal models existed [39]. Thus, strengthen further the importance of alternative to animal in BBB drug delivery studies.

### Live cells imaging in nanoparticle studies

Live cell imaging studies are other emerging *in vitro* techniques used in the study of nanoparticles and other related drugs [34]. With the live cell imaging systems, cellular activity like mode of death, proliferation and inhibition as a result nanoparticle or other compounds exposure can be studied. This method observe living cells; images are acquired from microscopes and other high content screening systems. The system gives a better view of cellular dynamics as it relates to any form of treatment and the researcher sees changes, as they are unfolding.

Cancer diagnosis and its therapy are among the areas that benefited from nanoparticles and live cell imaging systems studies [40]. Huge advances emerged through studying the dynamic biological processes taking place during cancer treatment [41,42]. This technique enabled researchers to follow the movement of individually labelled nanoparticles to specific parts of the cell. In some instances they are able to look at the receptor mediated transport and entrance of the particles into the chosen cells [34,40]. Two different types of nanoparticles (polystyrene and silica nanoparticles) were quantitatively analysed and localised in the cytoplasm and nucleus of Hep-2 cells about one hour after treatment using the live cell imaging [40]. Quantification was done through the fluorescence intensities of the tagged nanoparticles in the cytoplasm and nucleus of the cells. This observation has added to the list of nanoparticles with the capability of gene transfer into the nucleus.

### Nanoparticles in cancer screening

Over the last six decades or so more than 85,000 compounds were screened against cancers, most of which using short-term assays [43]. Cell and tissue culture (*in vitro*) studies were used as a baseline test in screening these compounds for their anti-cancer activity [43]. Significant number of the compounds were initially considered effective and promising, but failed clinical test in treating cancer [43]. By far the number of compounds that failed outweighs those that succeed to clinical usage [43]. The lost in time, money and more importantly lives, would likely wither if proper screening of intending anti-cancer agent were maximally utilized. One way of screening nanoparticles for possible anti-cancer activity is through *in vitro* methods using cells or tissues.

Cancer nanotechnology is emerging as result of interdisciplinary research and has great potential application in diagnosis and treating cancers [44]. As such, synthesis of hundreds of nano-biotechnology base cancer treatment/diagnostic tools is on the increase, this is to cater for cancer, a major public health problem [44,45]. Targeted drug deliveries in cancer treatment with reduce toxicity to surrounding normal cells and increase efficiency is but a few advantages explored while using this nanodelivery system [45]. Crossing the blood brain barrier to diagnose and treat CNS tumours, control release and increase solubility are also additional advantages of NDS [45]. Achieving these involves synthesizing and re-synthesizing several nanodelivery systems using nanotechnology, each time adjusting for a particular parameter to achieve the desired character. With each adjustment came the need for testing a peculiar property, doing this on animal’s means a lot in terms of time and money.

Nanoparticle in cancer treatment was studied using an *in vitro* model, where uptake of the polylactic-co-glycolic acid (PLGA) nanoparticles coated with polyvinyl alcohol (PVA) was shown to be 1.4 times less than that coated with vitamin E TPGS on a colorectal cancer cell line [45]. Recently our team demonstrated the effect of coating on iron oxide nanoparticle intercalated with Gallic acid in a cell culture-base study [46]. The magnetic nanoparticles with average size of 11.4 nm and coated with Polyethylene glycol (PEG) showed enhanced anti-cancer effect on breast cancer but not lung cancer compared to pure Gallic acid [46]. However, the same nanodelivery system containing Gallic acid coated with chitosan demonstrated lesser anti-cancer activity on both breast and colon cancer compare to the pristine Gallic acid [3]. Thus, cell culture studies allowed for the choice of better polymer on this nanoparticle containing Gallic acid in cancer treatment. It also helps in the screening of different cancer for which the delivery system will likely have better effect on.

The size-dependent quantum effect of nanoparticle is providing them unique physico-chemical and biological properties that makes them fundamentally different from their corresponding bulk counterpart, especially in cancer diagnosis and treatment [47]. *In vitro* cell proliferation assays were selected to show time and dose dependent the cytotoxicity of zinc oxide nanoparticles on colon cancer cells [47]. The same technique demonstrated the effect of particles sizes on the chosen cancer cells, where sizes below 30 nm showed higher toxicity compared to 90 nm size particles. The above-mentioned experiments look, sound relatively simple and cheap. However, the information generated could serve as a reasonable baseline in predicting possible *in vivo* outcome.

### Limitation of cell culture technique

Absence of immune effect, blood proteins, the endocrine system and the general lack of complex interaction of the whole animal in most *in vitro* system is limiting the potential transfer of some cell culture-based results of nanoparticle studies to animals/humans [48]. Tissue-specific differentiation functions of many cells and their physiological context of the primary cell cultures in *in vitro* systems are lost, like the loss of bio-transformational enzymes in primary liver cell cultures [49]. This lost in bio-transformational enzymes of liver cells will hinder the usage of primary liver cells in assessing nanoparticle for metabolism and toxicity potentials.

The assumption of neurotoxic potential of a compound if tested on neuronal cell line without considering the effect of blood brain barrier in animal model may lead to erroneous conclusion [48]. Recently, a group of researchers tried to evaluate the toxicity potential of zinc oxide nanoparticle on both cell and animal based models [50]. The two models showed a dose-dependent toxicity potential by the nanoparticle. However, in animal-based study, additional information emerged that relate route of administration and toxicity by the same delivery system. Another disturbing conclusion made from *in vitro* studies are that of glass fibre’s ability in generating reactive oxygen species and causing oxidant stress leading to DNA damage, but a low pathogenic potential was reported when tested in animal models [51]. *In vitro* study also reported kaolin to be as cytotoxic as quartz, and purified single-walled carbon nanotubes not inducing oxidative stress in cell studies but resulted in progressive interstitial fibrosis in mice [51]. In general, animal systems are extremely complicated, and the closer a proposed nanoparticle is for human use, the more it requires animal model research. However, properly planned *in vitro* technique will likely serve as a good screening mechanism for NDS. This will likely reduce cost, time and more importantly preserve more lives of animal in the future.

## Conclusion

There are many variables to consider when working with nanomaterial and these include type of material, their size, shape, surface, charge, coating, dispersion, agglomeration, aggregation, concentration and matrix. By carefully considering the experimental conditions, *in vitro* toxicity, bio-distribution, BBB deliveries, mechanism of cellular uptake and other related studies of nanoparticles can minimise the usage of *in vivo* system to assess these. This can play a role as a simple method to investigate the effect of such materials. However, animal systems are extremely complicated with unique bio-distribution, clearance, immune response, and metabolism. Therefore, *in vitro* study can only complement animal studies in assessing NDS. However, adequate and meaningful screening using cell culture technique will ensure reduction in the number of animal usage significantly.

## Competing interests

The authors declare that they have no competing interest.

## Authors’ contributions

AUK performed the data gathering and the initial write-up, SF, MZH and AP were involved drafting the manuscript, intellectual revision and gave approval for the final manuscript. All authors read and approved the final manuscript.
